# A Medical Decision Support System to Assess Risk Factors for Gastric Cancer Based on Fuzzy Cognitive Map

**DOI:** 10.1155/2020/1016284

**Published:** 2020-10-05

**Authors:** Seyed Abbas Mahmoodi, Kamal Mirzaie, Maryam Sadat Mahmoodi, Seyed Mostafa Mahmoudi

**Affiliations:** ^1^Department of Computer Engineering, Yazd Science and Research Branch, Islamic Azad University, Yazd, Iran; ^2^Department of Computer Engineering, Maybod Branch, Islamic Azad University, Maybod, Iran; ^3^Department of Electrical and Computer Engineering, Faculty of Sepideh Kashani, Birjand Branch, Technical and Vocational University (TVU), South Khorasan, Iran; ^4^Oral and Maxillofacial Pathology Department, School of Dentistry, Shahid Sadoughi University of Medical Sciences, Yazd, Iran

## Abstract

Gastric cancer (GC), one of the most common cancers around the world, is a multifactorial disease and there are many risk factors for this disease. Assessing the risk of GC is essential for choosing an appropriate healthcare strategy. There have been very few studies conducted on the development of risk assessment systems for GC. This study is aimed at providing a medical decision support system based on soft computing using fuzzy cognitive maps (FCMs) which will help healthcare professionals to decide on an appropriate individual healthcare strategy based on the risk level of the disease. FCMs are considered as one of the strongest artificial intelligence techniques for complex system modeling. In this system, an FCM based on Nonlinear Hebbian Learning (NHL) algorithm is used. The data used in this study are collected from the medical records of 560 patients referring to Imam Reza Hospital in Tabriz City. 27 effective features in gastric cancer were selected using the opinions of three experts. The prediction accuracy of the proposed method is 95.83%. The results show that the proposed method is more accurate than other decision-making algorithms, such as decision trees, Naïve Bayes, and ANN. From the perspective of healthcare professionals, the proposed medical decision support system is simple, comprehensive, and more effective than previous models for assessing the risk of GC and can help them to predict the risk factors for GC in the clinical setting.

## 1. Introduction

Gastric cancer (GC) which is one of the major cancers around the world with about one million new patients each year is known to be the third cause of cancer deaths [1, 2]. This represents an important public health issue in the world, especially in Central Asian countries, where the incidence of this disease is very high [2]. GC is a multifactorial disease, and its formation is related to various risk factors [3]. Various scientific methods, such as photofluorography and esophagogastroduodenoscopy, are used to diagnose GC in the early stages and can help reduce the mortality rate of GC with a practical approach [3]. Given that these methods are invasive and expensive, it is necessary to provide a simple inexpensive and effective tool for the diagnosis of people at risk for GC, which can then be followed by more accurate examinations. Moreover, appropriate prevention efforts can be made to reduce the incidence of this disease.

The initial definitions of the decision support system (DSS) consider it as a system to support decision-makers of the management in the semistructured and unstructured positions and decisions [4]. Accordingly, DSS means helping decision-makers and increasing their ability, not replacing their judgments [4]. Today, the use of DSSs has expanded in a variety of areas, such as management, industry, agriculture, information systems, medicine, and hundreds of other topics. The medical decision support system (MDSS) is a computer system designed to help physicians or other healthcare professionals in making clinical decisions. Some applications of the medical decision support system are outlined below [5]:

- Preventive care services, for example, screenings for blood pressure and cancer
- Patient symptom checker
- Care plan
- Guide to reducing long hospital stays
- Intelligent health monitoring systems

MDSS contains numerous advantages, of which the most important is to minimalize medical failure and make a relatively stable structure for diagnosing and treating the disease, thereby resolving various and conflicting ideas of specialists [5]. Therefore, it is vital to design and implement these models.

FCMs are regarded as soft computing methods that try attempting to act like humans for decision-making and reasoning [6]. In fact, an FCM is an instrument for modeling multifaceted systems, which is attained by integrating neural networks and fuzzy logic [7, 8], and to describe the complex system's performance utilizing concepts. This technique creates a conceptual model where each concept provides a characteristic or a state of a system dynamically interacting with these notions [9]. FCM is a graphical representation of a system structure [10]. According to the artificial intelligence, FCMs are dynamic learning networks; thus, more data to model the problem can help the system with adapting itself and reaching a solution. This conceptual model is not restricted to the exact measurements and quantities. Hence, it is very appropriate for concepts without accurate structures.

FCMs were presented by Kosko as a fuzzy directed graph with sign and feedback loops to illustrate the computational complexity and dependence of a model symbolically and explicitly [11]. In other words, a set of nodes is created by the FCM affecting each other via causal relations. The details and mathematical formulation of this technique are described in Supplementary Materials (available here). Using the benefits of fuzzy systems (if-then rules) and neural networks (teaching and learning), FCM was able to quickly prove its effectiveness in various areas so that we can see its successful presence in politics, economics, engineering, medicine, etc. [12].

In recent years, MDSS using FCM has been developed as one of the main applications of this tool. FCM has emerged as a tool for representing and studying the behavior of systems, and it can deal with complex systems using an argumentative process. This study is aimed at providing an MDSS for assessing the risk of GC using FCM.

In the following, some successful instances of FCM applications regarding decision support systems are provided. Papageorgiou et al. [13] utilized FCM for predicting infectious diseases and infection severity. A novel FCM-based technique was presented by Amirkhani et al. [14] to screen and isolate UDH from other internal brain lesions. Hence, they examined 86 patients in Shahid Beheshti Hospital in Isfahan City. The pathologist extracted the ten key properties needed to screen these lesions to use them as the key concepts of FCM. The accurateness of the suggested technique was 95.35%. Based on the results, it was indicated that not only the suggested FCM contained a high accuracy level it is also able to preset an acceptable false-negative rate (FNR). A decision support system was proposed by Baena de Moraes Lopes et al. [7] to diagnose the changes in urinary elimination, based on the nursing terminology of North American Nursing Diagnosis Association International (NANDA-I). For 195 cases of urinary incontinence, an FCM model was utilized after the NANDA-I classifications. The high specificity and sensitivity of 0.92 and 0.95, were, respectively, found by the FCM model; however, a low specificity value was provided in the determination of the diagnosis of urge urinary incontinence (0.43) along with a low sensitivity value to overall urinary incontinence (0.42).

Recently, the use of FCM with Hebbian-based learning capabilities has increased. According to [15], a decision-making framework was proposed that can accurately assess the progression of depression symptoms in the elderly people and warn healthcare providers by providing useful information for regulating the patient's treatment. According to [16], a risk management system for familial breast cancer was presented using the NHL-based FCM technique. Data needed for this study were extracted from 40 patients and 18 key features were selected. The results showed that the accuracy is 95%. According to [17], the first specialized diagnostic system for obesity was proposed based on psychological and social characteristics. In this study, a mathematical model based on FCM was presented. According to the proposed model, the effects of different weight-loss treatment methods can be studied.

No certain reason exists for GC. The cause-effect associations are not systematically investigated and understood so far between the integrated impacts of the multiple risk factors on the probability of developing GC. Even the ideas of radiologists and oncologists are greatly subjective in this regard. In such instances, it is considered to use an FCM as a human-friendly and transparent clinical support instrument to determine the cause-effect associations between the factors and the subjectivity can be remarkably eliminated by the degrees of its effects on the risk level. The present work is mainly focused on developing a clinical decision-making instrument in terms of an FCM to evaluate GC risk.

## 2. Methods

### 2.1. FCM Model for GC Risk Factors

Addressing GC is a complex process that needs to understand the various parameters, risk factors, and symptoms to make the right decision and assessment. This study assesses the risk of GC by providing a medical decision-making system. The design of this decision-making system is based on a proposed model of FCM, which is presented below. Designing and developing a suitable FCM require human knowledge to describe a decision support system. In this study, GC specialists are used for the development of the FCM model. The development of the FCM model is divided into three main steps, which is briefly summarized:

1. Identify concepts
2. Determine the relationships between concepts and initial weights
3. Weighting

First, the experts individually identify the factors that contribute to GC. In the following, common concepts among specialists are selected as model nodes. The second step is to identify the relationships between concepts. To this end, experts define the interactions between concepts with respect to fuzzy variables. To do so, determine the relationship and the direction of the relationship (if any). The amounts of these effects are expressed as very low, low, medium, high, and very high. Finally, the linguistic variables expressed by the experts are integrated. Using the SUM technique, these values are aggregated and the total linguistic weight is generated by the “centric” defuzzification method and converted to a numerical value. The corresponding weight matrix is then constructed. Choosing a learning algorithm to teach initial weights is the third step of this method. The purpose of a learning algorithm, setting the initial weight, is the same way as neural networks to improve the modeling FCM.

To better understand, these steps were used step by step to develop an FCM model for GC. For this purpose, the opinions of three specialists were used. In the first phase of the research presented in this article, information on GC risk factors was collected from medical sources, pathologists, and informal sources [18–48]. The collected knowledge was transformed into a well-structured questionnaire and presented to three experts. The questionnaire includes risk factors associated with GC. According to three experts, 27 common features were identified as the major risk factors for end-stage GC. To better understand, we used the mentioned process step by step to develop an FCM model for GC.

**Figure 1:**
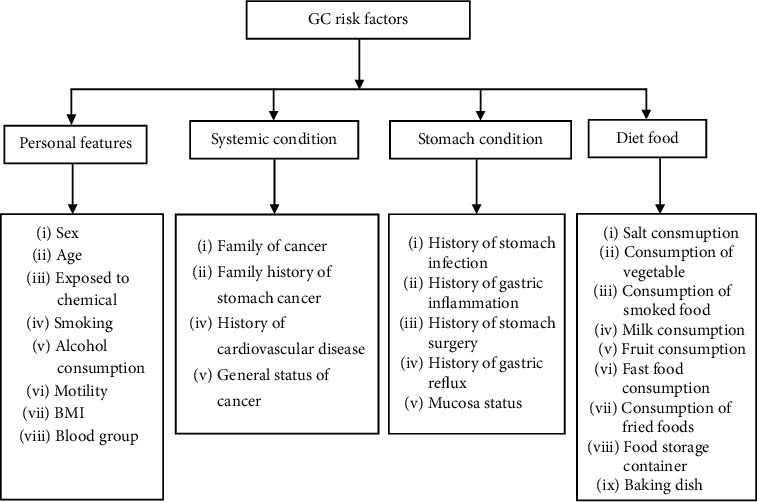
Classification of GC risk factors.

Risk factors for gastric cancer may be categorized into four groups (personal features, systemic conditions, stomach condition, and diet food), each of which includes several risk factors. The final features are presented in Figure 1, and their explanations are given in Table 1.

In the second phase, first, the sign for the relationship between the two concepts is determined, and finally, the numerical values of the two concepts are calculated. Five membership functions were used for this purpose. Consider the following example.

*1^st^ specialist*: C4 has a great impact on C27.

*2^nd^ specialist*: C4 has a moderate impact on C27.

*3^rd^ specialist*: C4 has a great impact on C27.

**Figure 2:**
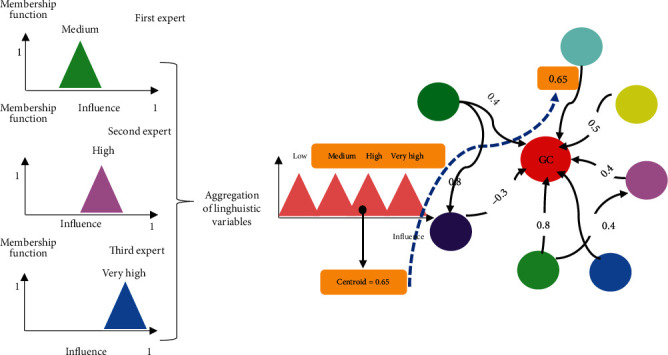
Aggregation and defuzzification of linguistic weights.

Using the SUM method, the above three linguistic weights (high, very high, and very high) are aggregated. The above three linguistic weights (high, very high, and very high) are aggregated using the SUM method.  Figure 2 represents the centroid defuzzification method that is implemented to calculate the numerical value of the weight in the range [−1, 1].

**Figure 3:**
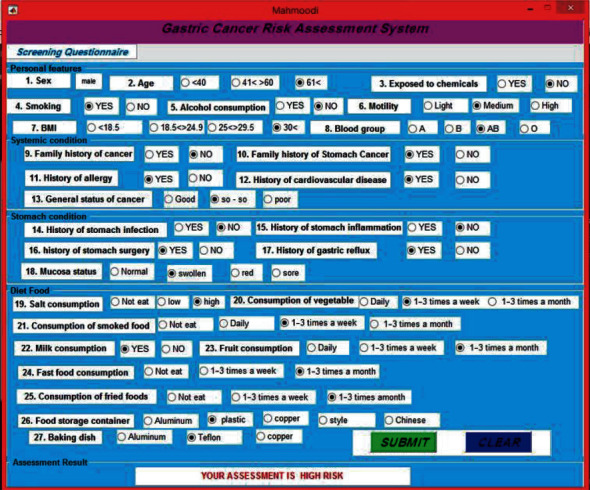
User interface of the proposed MDSS.

Using this method, the weight of all relationships between the concepts related to FCM for GC was calculated. The developed FCM is shown in Figure 3. In the third step, we used a learning algorithm to train the model, which includes updating the relationship weight, and finally, a fuzzy cognition map for GC risk factors was extracted. For this purpose, data collected from 560 patients referred to Imam Reza Hospital in Tabriz (after the preprocessing steps) were used through a questionnaire.  Table 2 shows the features, values, and frequency of patients.

**Figure 4:**
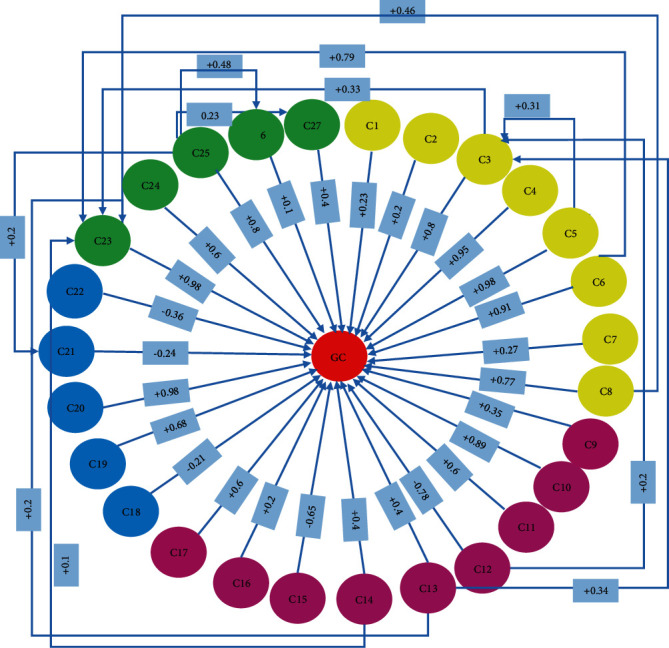
FCM model for GC risk factors.

Figure 4 shows the proposed FCM model for risk factors of GC. This FCM has 28 concepts and 38 edges with their weights. Considering the 28 concept nodes, 27 are the ultimate physician-selected features that interfere with the disease and are shown by the values C1 to C27. The central node is the concept of GC, which receives and collects interactions from all other nodes. The positive weight of an edge indicates that it has a positive effect on the incidence of GC, and the negative weight indicates the role of deterrence in the incidence of the disease. The yellow, purple, blue, and green colors were used to specify the category of any feature or concept. The C1 to C8 features specified with yellow were classified as personal features. The violet color was used for the C9 to C17 features of the diet food category. Blue and green were also used for the C18 to C22 features of the systemic condition, respectively, and C23 to C27 features were used for the stomach condition category.

### 2.2. Learning FCM Using NHL Algorithm

GC specialists were well positioned to create FCM in our method. Nonlinear Hebbian Learning (NHL) is utilized to learn the weights due to no access to a relatively large data set, causal weight optimization, and more accurate results [49]. The Hebbian-based algorithms were used for FCM training to determine the best matrix in terms of expert knowledge [50]. Algorithms set the FCM weights through existing data and a learning formula in terms of repetition and Hebbian rule methods [50]. The NHL algorithm is based on the assumption that all of the concepts of the FCM model are stimulated at each time step and their values change. The value *ω*_*ji*_ corresponding to the concepts of *c*_*j*_ and *c*_*i*_ is updated, and the weight *ω*_*ji*_ is corrected in iteration *k*. The value of *A*_*i*_^(*k* + 1)^ is determined in the (*k* + 1)th iteration. The impact of concepts with values *A*_*j*_ and corrected weighted values *ω*_*ij*_^(*k*)^ in iteration *k* is determined by

(1) $$\begin{array}{c} A_{i}^{\left(k+1\right)}=f\left(A_{i}^{\left(k\right)}+\overset{n}{\underset{j=1,i\neq j}{\sum }}\omega_{ji}^{\left(k\right)}\cdot A_{j}^{\left(k\right)}\right). \end{array}$$

Each of the concepts in the FCM model may be input or output concepts. A number of concepts are defined as output concepts (OCs). These concepts are the state of the system in which we want to estimate the value that represents the final state of the system. The classification of concepts as input and output concepts is by the experts of the group and according to the subject under consideration. The mathematical relations used in the NHL algorithm for learning FCM are shown in equations (1) and (2).

(2) $$\begin{array}{c} {\Delta \omega }_{ji}=\eta_{t}A_{i}^{\left(K-1\right)}\left(A_{j}^{\left(K-1\right)}-\omega_{ji}^{\left(K-1\right)}A_{i}^{\left(K-1\right)}\right), \end{array}$$

where *η* is a scaling parameter called the learning rate. *η* is a very small positive scaler factor called learning parameter. Its value is obtained through test error.

(3) $$\begin{array}{c} \omega_{ji}^{\left(k\right)}=\gamma \cdot \omega_{ji}^{\left(k-1\right)}+\eta A_{i}^{\left(k-1\right)}\left(A_{j}^{\left(k-1\right)}-\mathrm{sgn}\left(\omega_{ji}\right)\omega_{ji}^{\left(k-1\right)}A_{i}^{\left(k-1\right)}\right). \end{array}$$

Equation (3) is the main equation of the NHL algorithm. *γ* is the weight decay parameter. The values of concepts and weights *ω*_*ji*_^(*k*)^ are calculated by equations (1) and (3), respectively. In fact, the NHL algorithm updates the basic matrix nonzero elements suggested by the experts in each iteration. The following criteria determine when the NHL algorithm ends [50].

(a) The terminating function *F*1 is given as

(4) $$\begin{array}{c} F1=\left\|{\text{OC}}_{i}-T_{i}\right\|, \end{array}$$

where *T*_*i*_ is the mean value of OC_*i*_.

This kind of metric function is suitable for the NHL algorithm used in the FCMs. In each step, *F*1 calculates the Euclidean distance for OC_*i*_ and *T*_*i*_. Assuming that OC_*i*_ = [*T*_*i*_^min^, *T*_*i*_^max^], *T*_*i*_ is calculated by

(5) $$\begin{array}{c} T_{i}=\frac{T_{i}^{\min}+T_{i}^{\max}}{2}. \end{array}$$

Given that the FCM model has *m*-OCs, for calculating *F*1, the sum of the square between *m*-OCs and *m*‐*T*_*s*_ can be calculated by

(6) $$\begin{array}{c} F1=\sqrt{\overset{m}{\underset{j=1}{\sum }}{\left({\text{OC}}_{i}-T_{i}\right)}^{2}}. \end{array}$$

After *F*1 is minimized, the situation ends. b) The second condition for completing the algorithm is the difference between two consecutive OCs. This value should be less than *e*. Therefore, the value of the (*k* + 1)th iteration should be less than *e* based on

(7) $$\begin{array}{c} F2=\left|{\text{OC}}_{i}^{\left(t+1\right)}-{\text{OC}}_{i}^{\left(t\right)}\right|<e=0.002. \end{array}$$

In this algorithm, the values of the parameters *η* and *γ* are determined through test error. After several tests, the values of *η* and *γ* show the best performing algorithm. Finally, when the algorithmic termination conditions are met, the final weight matrix (*ω*_NHL_) is obtained.

For the convenience of end-users, a graphical interface is designed using the GUI in MATLAB for the proposed system. The user interface for the GC risk prediction software is shown in Figure 3.

For example, the user enters the requested information into the system. The system displays the risk assessment result after receiving information from the user and using the proposed NHL-FCM model.

For the comparison of classification accuracy, the same data set is used for classification with other machine learning models. Backpropagation neural network, support vector machine, decision tree, and Bayesian classifier were used in the Weka toolkit V3.7 to test other learning algorithms. For this purpose, the Excel file containing the collected data collection was converted to .arff format so that it can be read for Weka. Then, the required steps for data preprocessing were performed. In this software, one of the most common methods of evaluating the performance of categories that divide the tagged data set into several subsets is cross-validation. 10-fold cross-validation was used for all the studied algorithms. 10-fold cross-validation divides the data set into 10 parts and performs the test 10 times. In each step, one part is considered as a test and the other 9 parts are considered for training. In this way, each data is used once for testing and 9 times for training. As a result, the entire data set is covered for training and testing.

The backpropagation neural network with 27 input neurons, 10 neurons, and 3 output nodes was used as the multilayer perceptron. Also, for classification of the assess risk into three classes, high, medium, and low, the support vector machine, decision tree C4.5, and Naïve Bayesian classifier were used.. Given that the data studied are not linearly separable, we need to use the core technology to implement the SVM algorithm. The core technology is one of the most common techniques for solving problems that are not linearly separable. In this method, a suitable core function is selected and executed. In fact, the purpose of kernel functions is to linearize nonlinear problems. There are several kernel functions in Weka. The RBF (Radial Basis Function) was used to run the SVM algorithm. By selecting and running the C4.5 algorithm, you can see the results of the classification. Also, the tree created by this algorithm can be seen graphically, which is a large tree. The three categories of high risk, medium risk, and low risk were selected as target variables and other characteristics as predictive variables. The leaves of the tree are the target variables and can be seen as a number of rules according to the model made by the tree. Naïve Bayesian was another classification algorithm that was implemented using Weka on the studied data, and its results were examined. This algorithm uses a possible framework to solve classification problems.

## 3. Results

To analyze the performance of the proposed method, we divided the data into two categories. The proposed model was trained using 70% of the patient records (392 records) based on the NHL algorithm and tested using 30% of the records (168 records). Considering 168 patient records selected for testing randomly, there were 56 records in the high category, 64 records in the medium category, and 48 records in the low category.

Root square error (RMSE) and performance measure accuracy, recall, precision, and mean absolute error (MAE) are the key behavior measures in the medical field [17] widely utilized in the literature. To determine accuracy, recall, and precision, the turbulence matrix was utilized. A confusion matrix is a table making possible to visualize the behavior of an algorithm.  Table 3 represents the general scheme of a confusion matrix (with two groups C1 and C2).

The matrix contains two columns and two rows specifying the values including the number of true negatives (TN), false negatives (FN), false positives (FP), and true positives (TP). TP shows the number of specimens for class C1 classified appropriately. FP represents the number of specimens for group C2 classified inaccurately as C1. TN shows the number of samples for class C2 classified correctly. FN represents the number of specimens for class C1 classified incorrectly as class C2.

- *Accuracy*: accuracy represents the ratio of accurately classified specimens to the total number of tested samples. It is determined by

(8) $$\begin{array}{c} \text{Accuracy}=\frac{\left(\text{TN}+\text{TP}\right)}{\left(\text{TN}+\text{TP}+\text{FN}+\text{FP }\right)}. \end{array}$$

(ii) *Recall*: recall is the number of instances of the class C1 that has actually predicted correctly. It is calculated by

(9) $$\begin{array}{c} \text{Recall}=\frac{\text{TP}}{\text{TP}+\text{FN}}. \end{array}$$

(iii) *Precision*: it represents the classifier's ability not to label a C2 sample as C1. It is calculated by

(10) $$\begin{array}{c} \text{Precision}=\frac{\text{TP}}{\text{TP}+\text{FP}}. \end{array}$$

The MAE performance index is calculated by

(11) $$\begin{array}{c} \text{MAE}=\frac{1}{N}\left(\overset{N}{\underset{L=1}{\sum }}\overset{C}{\underset{J=1}{\sum }}\;\left|{\text{OC}}_{JL}^{\text{Real}}-{\text{OC}}_{JL}^{\text{Predicted}}\;\right|\right). \end{array}$$

In equation (11), *N* represents the number of training data (*N* = 560), *C* shows the number of output concepts (*C* = 3), and  OC_*L*_^Real^ − OC_*L*_^Predicted^ denotes the difference between the *l*th decision output concept (OC) and its equivalent real value (target) by appearing the *k*th set of input concepts to the input of the tool.

The RMSE evaluation index is defined based on

(12) $$\begin{array}{c} \text{RMSE}=\sqrt{\frac{1}{NC}\left(\overset{N}{\underset{L=1}{\sum }}\overset{C}{\underset{J=1}{\sum }}{\left({\text{OC}}_{JL}^{\text{Real}}-{\text{OC}}_{JL}^{\text{Predicted}}\right)}^{2}\;\right)}, \end{array}$$

where *N* is the number of training sets and *C* is the system outputs.

Table 4 shows the accuracy results obtained from the proposed method and other standard categorizers. The proposed method works better than other categories because of the efficiency of the NHL's efficiency for working with very small data to correct FCM weight. As a result, optimal decisions are made for output concepts.

The results show that the highest total accuracy is related to the proposed method (95.83%) which is about 5% higher than the accuracy of the MLP-ANN algorithm. The highest precision and recall are related to the proposed algorithm, which are, respectively, 96.77% (medium) and 98.21% (high). It also shows that the training error of the proposed method based on NHL is less than the other algorithms used in this study.

As stated, *γ* and *η* are two learning parameters in the NHL algorithm. In this algorithm, the upper and lower limits of these parameters are determined by trial and error in order to optimize the final solution. After several simulations with parameters *γ* and *η*, it was observed that the use of large amounts of *γ* causes significant changes in weights and weight marks. Also, simulation with small *η* also creates significant weight changes, thus preventing the weight of concepts from entering the desired range. For this reason, values *γ* and *η* are limited to 0 < *γ* < 0.1 and 0.9 < *η* < 1. In each study, a constant value is considered for these parameters.

After several investigations, it was found that the best performance of the category is related to *η* = 0.045 and *γ* = 0.98. The classification results obtained for the different values of learning parameters are presented in Table 5.

## 4. Discussion

In this study, we designed a risk prediction model and a GC risk assessment tool using data from a study on a population of patients referring to the gastroenterology unit of Imam Reza Hospital in Tabriz. The proposed model presented in this study is attempting to rationalize beyond the analyses of clinical experts and increase the ability of experts to make logical decisions in a clinical setting for patients with different levels of risk factors for GC and help clinical specialists to make a logical decision about optimal preventive methods for patients.

The 95.8% overall classification accuracy obtained through the Hebbian-based FCM using 560 patients indicates a high level of coordination between the proposed system and medical decisions, and the proposed decision support tool can be trusted for clinical professionals and also helps them in the process of risk assessment of gastric GC.

Specifically, our risk assessment tool is simple and inexpensive to use in the clinical environment, because many other methods to predict the risk of GC are invasive. Therefore, this is an effective instrument for estimating the population at risk of cancer in the future. The results show that this new model can predict the probability of developing GC concerning the characteristics specified in this study with a better accuracy than previous studies.

In recent years, several researches have been carried out on the development and validation of risk assessment tools for various cancers [51, 52]. Recent studies have shown that the combination of *H. pylori* antibody and serum pepsinogen can be a good predictor of GC [53, 54].

We believe that only two other evaluation instruments exist for GC rather than ours. Based on the Japan Public Health Center-based Prospective Study, a device was designed to estimate the cumulative probability of GC incidence including sex, age, smoking status, the mixture of *H. pylori* antibody and serum pepsinogen, consumption of salty food, and family history of GC as the risk factors [55]. A good performance was found by the model based on calibration and discrimination. Based on [2], a risk evaluation instrument for GC was proposed in the general population of Japan. In this work, gender, age, the combination of *Helicobacter pylori* antibody and pepsinogen status, smoking status, and hemoglobin A1C level were risk factors for GC.

The risk factors chosen in these two studies were very limited to a few specific characteristics and had little similarity to the factors in our study. Risks such as consumption of fruits and vegetables, alcohol consumption, history of cardiovascular disease, blood type, milk consumption, history of allergy, gastric reflux, storage containers, food intake, and family history of cancer did not exist in both studies in spite of their importance in previous studies. Factors such as salt intake and a history of GC are known as causes of GC that did not exist in [2]. Another remarkable point in our study is that, given the nature of the proposed model, this method addresses the effects of factors that are sometimes related to each other or even the mutual effects that might put each other at risk, but it is not included in the two previous studies.

Another advantage of the proposed method than other algorithms is that other methods cannot provide any explicit causal relationship and the system works as a black box. This problem also makes these algorithms less suited to medical decision support systems. Finally, the new system has the following benefits:

- It examines the factors that have not been taken into account in previous models to assess the risk of GC
- Because of the use of new factors, this model can be more effective in predicting the risk of GC
- The proposed model is presented by a software that has a simple, convenient, and user-friendly interface
- The use of this software by physicians and other researchers can tackle individual healthcare decisions
- It helps healthcare professionals decide on individual risk management mechanisms

The system presented in this study has the following limitations: (1) a small sample of patients used to learn and anticipate GC, (2) the heavy dependence of this model on knowledge of domain specialists, (3) dependence on initial conditions and communication, and (4) the absence of external validation of the forecast system. Although this system has nice results due to the use of an appropriate database and the important and relevant GC factors, the generalizability of our results cannot be proved without the experiment of the system in another data set. As a result, it is necessary to use a larger statistical population to test the proposed model.

## 5. Conclusions

Assessing the level of risk for GC is very important and helps make decisions about screening. Given the limited number of GC risk assessment tools that have been proposed so far, there is no tool that comprehensively covers the risk factors in scientific studies on GC. The proposed model based on soft computing covers all the factors influencing the incidence of GC. The classification accuracy of the proposed method is higher than other methods of the machine learning classification, such as the decision tree and SVM. This is due to the useful features of FCM for checking domain knowledge and determining the initial structure of FCM and the initial weights and then using the NHL algorithm to teach the FCM model and adjust these weights. The FCM-based model is comprehensive, transparent, and more effective than previous models for assessing the risk of GC. As a result, this risk assessment tool can help diagnose people with a high risk of GC and help both healthcare providers and patients with the decision-making process. Our future work is to use more features and variations and other learning algorithms to determine the weight of the edges in the FCM.

## Figures and Tables

**Table 1:** Risk factors of GC.

Risk factors	Description
C1: sex	Studies show that men around the world are diagnosed with GC almost twice as much as women [18].
C2: blood group	Scientific research shows that there is a significant relationship between blood type and GC. The blood groups A and O have the highest and lowest incidence of GC, respectively [19].
C3: BMI	High BMI increases GC [20]. In 2016, the IACR formed a team of specialists. They reported that GC is one of the diseases caused by excessive fat gain and high BMI [21].
C4: age	The risk of GC increases with age [18, 22, 23].
C5: motility	People with any regular physical activity have a lower risk of GC than nonactive people. According to the US Physical Activity Guidelines Advisory Committee (2018), moderate evidence showed that physical activity reduces the risk of various cancers, including GC [21].
C6: alcohol consumption	Regular alcohol consumption increases the risk of GC [24, 25].
C7: exposed to chemicals	Some jobs exposed to chemicals, such as cement and chromium, increase the risk of GC [26].
C8: smoking	Smoking increases the risk of GC [27, 28].
C9: salt consumption	High salt intake increases the risk of GC [23, 29, 30].
C10: consumption of vegetable	The daily consumption of 200-200 grams of vegetables per day may reduce the risk of GC [31].
C11: consumption of smoked food	The smoked food is a great source of polycyclic aromatic hydrocarbons (PAHs). Scientific research has shown that this biopollutant is one of the factors involved in many cancers, including GC [32, 33].
C12: milk consumption	Increasing dairy consumption, such as milk, is associated with a lower risk of GC [34].
C13: fast food consumption	Fast food consumption is one of the factors affecting the incidence of GC [35].
C14: consumption of fried foods	The results of scientific studies show that people who use a lot of fried foods in their diet are at increased risk of GC [27, 28].
C15: fruit consumption	A daily consumption of 120-150 grams of fruit per day may reduce the risk of GC [31].
C16: food storage container	Today's food containers are often made of chemicals, such as plastics that contain bisphenol A. Thus, it can be the source of various types of cancer and hormonal disorders [36].
C17: baking dish	The use of metal containers, such as aluminum for cooking, can be a factor in the development of diseases because these types of metals, when exposed to heat, emit a small amount of lead [37].
C18: history of allergy	Recent studies indicate that the history of allergic diseases is associated with a lower risk of GC [38].
C19: family history of cancer	A family history of cancer in certain specific sites may be associated with a risk of GC [39].
C20: family of GC	This risk factor is strongly associated with different types of GC [40, 41].
C21: history of cardiovascular disease	People with cardiovascular disease are at a lower risk of GC because of using some drugs [42].
C22: general status of cancer	People with a good general health status are less likely to be at risk of GC [43].
C23: history of gastric reflux	Gastric reflux causes a 3-10% percent increase in being at risk of GC [44].
C24: history of stomach surgery	Gastric surgeries, such as gastric ulcers, may increase the risk of cancer [45].
C25: history of stomach infection	Helicobacter pylorus is the most important risk factor for GC [46–48].
C26: mucosa status	Gastric ulcers are considered as a risk factor for GC [35].
C27: history of gastric inflammation	The history of gastric inflammation is one of the most important factors in the incidence of GC [35].

**Table 2:** Data sets.

Features	Range	Number	Percent
Sex	Male	256	45.7%
	Female	304	54.3%
Age	<40	20	3.47%
	41–60	210	37.5%
	≥61	330	59.03%
Blood group	A	123	21.96%
	B	78	13.92%
	AB	80	14.28%
	O	279	49.82%
BMI	BMI > 30	69	12.32%
	25 < BMI > 29.5	76	13.57%
	18.5 < BMI > 24.9	120	21.42%
	BMI < 18.5	293	52.32%
Motility	Light	156	27.85%
	Medium	236	42.14%
	High	168	30%
Alcohol consumption	Yes	85	15.17%
	No	475	84.82%
Exposed to chemicals	Yes	54	9.64%
	No	506	90.35%
Smoking	Yes	198	35.35%
	No	362	64.64%
Salt consumption	None	10	1.78%
	Low	175	31.25%
	High	375	66.96%
Consumption of vegetable	Daily	26	4.64%
	1-3 times a week	214	38.21%
	1-3 times a month	320	57.14%
Consumption of smoked food	None	5	0.89%
	Daily	0	0%
	1-3 times a week	149	26.60%
	1-3 times a month	406	72.5%
Milk consumption	Yes	214	38.21%
	No	346	61.78%
Fast food consumption	None	4	0.71%
	1-3 times a week	315	56.25%
	1-3 times a month	241	43.03%
Consumption of fried foods	None	0	0%
	1-3 times a week	191	34.10%
	1-3 times a month	369	65.89%
Fruit consumption	None	6	1.07%
	1-3 times a week	185	33.03%
	1-3 times a month	369	65.89%
Food storage container	Aluminum	216	38.57%
	Plastic	301	53.75%
	Copper	32	5.71%
	Style	9	1.60%
	Chinese	2	0.35%
Baking dish	Aluminum	10	1.78%
	Teflon	390	69.64%
	Copper	21	3.75%
History of allergy	Yes	89	15.89%
	No	471	84.10%
Family history of cancer	Yes	211	37.67%
	No	349	62.32%
Family of GC	Yes	123	21.965
	No	437	78.03%
History of cardiovascular disease	Yes	185	33.03%
	No	375	66.96%
General status	Good	79	14.10%
	So-so	190	33.92%
	Poor	291	51.965
History of gastric reflux	Yes	234	41.78%
	No	326	58.21%
History of stomach surgery	Yes	48	8.57%
	No	512	91.42%
History of stomach infection	Yes	176	31.42%
	No	384	68.57%
Mucosa status	Normal	94	16.78%
	Swollen	126	22.5%
	Red	157	28.03%
	Sore	183	32.67%
History of gastric inflammation	Yes	163	29.10%
	No	397	70.89%
Risk score	High	300	53.57%
	Moderate	186	33.21%
	Low	74	8.39%

**Table 3:** Confusion matrix.

	Predicted class		
Actual class		C1	C2
	C1	True positive (TP)	False positive (FP)
	C2	False negative (FN)	True negative (TN)

**Table 4:** Performance metrics.

Classifiers	+	High	Medium	Low	Class recall	Class precision	Overall accuracy	RMSE	MAE
Decision trees	High	30	10	1	53.57	73.17	76.78	0.5120	0.721
	Medium	16	52	0	81.25	76.47			
	Low	10	2	47	97.91	79.66			
Naïve Bayes	High	40	8	5	71.42	75.47	80.35	0.334	0.645
	Medium	8	56	4	87.5	77.77			
	Low	8	0	39	81.25	82.97			
SVM	High	46	2	4	82.14	88.46	86.9	0.193	0.342
	Medium	0	60	4	93.75	93.75			
	Low	10	2	40	83.3	76.92			
MLP-ANN	High	49	2	7	87.5	84.48	90.47	0.248	0.097
	Medium	4	58	4	90.62	87.87			
	Low	3	4	45	93.75	86.53			
Proposed model	High	55	1	1	98.21	96.49	95.83	0.173	0.0471
	Medium	1	60	1	93.75	96.77			
	Low	0	3	46	95.83	93.87			

**Table 5:** Classification results, based on different values of *η* and *γ*.

*η*	*γ*	Confusion matrix			Classification accuracy (%)
		High	Medium	Low	
0.01	0.97	50	4	7	88.69
		4	59	1	
		2	1	40	
0.03	0.95	45	6	1	89.28
		5	58	0	
		6	0	47	
0.045	0.98	55	1	1	95.83
		1	60	1	
		0	3	46	
0.05	0.96	54	6	0	94.04
		1	56	0	
		1	2	48	
0.055	0.96	53	2	5	91.6
		2	58	0	
		1	4	43	

## Data Availability

The data used to support the findings of this study are available from the corresponding author upon request.

## Supplementary Materials

Supplementary Materials A review of fuzzy cognitive maps [56].
