# Supplementary material for: A Medical Decision Support System to Assess Risk Factors for Gastric Cancer Based on Fuzzy Cognitive Map
Source: Comput Math Methods Med. 2020 Oct 5;2020:1016284. doi: 10.1155/2020/1016284 (PMC7556058; doi:10.1155/2020/1016284)
Supplement: Supplementary Materials — A review of fuzzy cognitive maps [56]. [file 1016284.f1.pdf]

Supplementary Materials

A Review of Fuzzy Cognitive Maps

Formally, FCM includes a set of concepts, each represented by C_i, i = 1, ..., N (N shows the overall number of concepts) affecting each other through the cause and effect relations[11]. A key major is represented by each concept, which is considered by a value of $A_i \in [0,1]$. These concepts are connected by the weighted arcs represented by their relationships. Figure S1 represents an FCM.

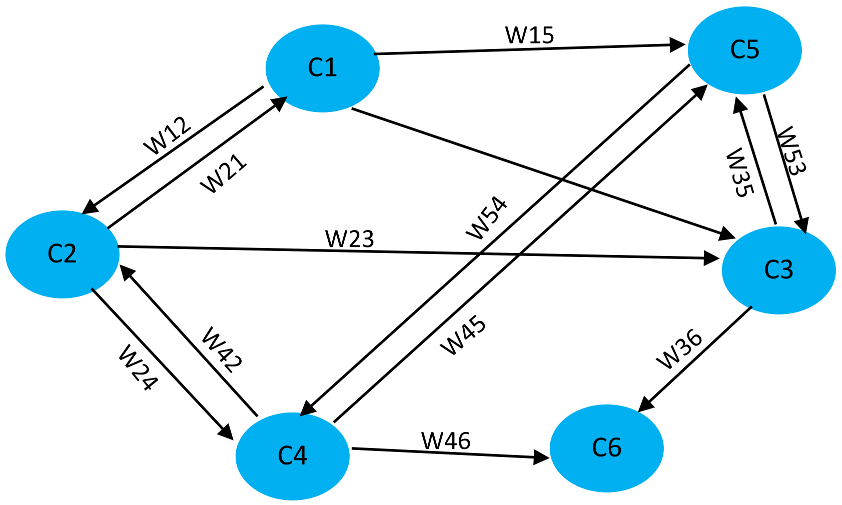

FIGURE S1: A Simple FCM.

Each relation is within two concepts C_i and C_j possessing a W_{ij} weight. W_{ij} represents the power of their association. These weights are attained by converting the fuzzy values provided by experts to numerical values. The sign W_{ij} shows whether the association between the two concepts is inverse or direct. Hence, 3 kinds of weight exist:

$$\begin{cases} Wij > 0 \text{ ; positive association} \\ Wij = 0 \text{ ; no association} \\ Wij < 0 \text{ ; negative association} \end{cases}$$

Utilizing the system experience human and knowledge, it is possible to determine the number and type of nodes and the initial FCM weight. By giving the values to the weights and concepts,

the FCM is converged to a stable state. At each stage (k), each concept's value (A_i) is influenced by the other nodes attached to it and is updated in terms of Eq.13[11].

$$A_i^{(k+1)} = f\left(A_i^{(k)} + \sum_{j=1, i\neq j}^{N} A_j^{(k)} W_{ji} \right) \tag{13}$$

Where $A_i^{(k)}$shows the value of the concept C_i at step K. $A_j^{(k)}$ represents the value of the concept C_j at step K. W_{ji} shows the connection weight between the concept C_j and C_i and F denotes a threshold function compressing the outcome in an interval [0,1], which can be provided as various kinds of functions like Bivalent trivalent hyperbolic tangent and sigmoid. These functions are explained as follows (Eq.12 to Eq.17)[11]:

$$f_{\text{sign}}(x) = \begin{cases} 0 & x \leq 0 \\ 1 & x > 0 \end{cases} \tag{14}$$

$$f_{\text{tri}}(x) = \begin{cases} -1 & x \leq -0.5 \\ 0 & -0.5 < x > 0.5 \\ +1 & x \geq 0/5 \end{cases} \tag{15}$$

$$f(x) = \tanh(x)\ \frac{e^{x} - e^{-x}}{e^{x} + e^{-x}} \tag{16}$$

$$f(x) = \frac{1}{1 + e^{-ax}} \qquad a \geq 0 \tag{17}$$

In Eq.17, α represents the convergence degree in the sigmoid function.

In simple uses with fewer concepts, an FCM is normally created manually. Nevertheless, it is not possible to use this technique in real applications due to numerous variables. Data-driven algorithms known as learning algorithms for FCM modeling are utilized for solving this problem to make and train FCM.

Three main ways exist to train FCM including population-oriented approaches, adaptive techniques (normally based on the Hebbian law), and the hybrid approaches combining the population-based and adaptive methods [57]. Hebbian-based learning algorithms are regularly utilized for medical decision-making support, in which early information of experts is available to model using a relatively small set of historical data to classify and predict.
